# South African Breast Cancer and HIV Outcomes Study: Methods and Baseline Assessment

**DOI:** 10.1200/JGO.2015.002675

**Published:** 2016-06-22

**Authors:** Herbert Cubasch, Paul Ruff, Maureen Joffe, Shane Norris, Tobias Chirwa, Sarah Nietz, Vinay Sharma, Raquel Duarte, Ines Buccimazza, Sharon Čačala, Laura W. Stopforth, Wei-Yann Tsai, Eliezer Stavsky, Katherine D. Crew, Judith S. Jacobson, Alfred I. Neugut

**Affiliations:** **Herbert Cubasch**, **Sarah Nietz**, **Paul Ruff**, **Maureen Joffe**, **Shane Norris**, **Tobias Chirwa**, **Vinay Sharma**, and **Raquel Duarte**, University of the Witwatersrand; **Sarah Nietz**, Charlotte Maxeke Johannesburg Academic Hospital, Johannesburg; **Herbert Cubasch** and **Vinay Sharma**, Chris Hani Baragwanath Academic Hospital, Soweto; **Ines Buccimazza**, University of KwaZulu-Natal; **Ines Buccimazza**, Inkosi Albert Luthuli Central Hospital, Durban; **Sharon Čačala** and **Laura W. Stopforth**, Grey’s Hospital, Pietermaritzburg, South Africa; and **Wei-Yann Tsai**, **Eliezer Stavsky**, **Katherine D. Crew**, **Judith S. Jacobson**, and **Alfred I. Neugut**, Columbia University, New York, NY.

## Abstract

**Purpose:**

In low- and middle-income, HIV-endemic regions of sub-Saharan Africa, morbidity and mortality from the common epithelial cancers of the developed world are rising. Even among HIV-infected individuals, access to antiretroviral therapy has enhanced life expectancy, shifting the distribution of cancer diagnoses toward non–AIDS-defining malignancies, including breast cancer. Building on our prior research, we recently initiated the South African Breast Cancer and HIV Outcomes study.

**Methods:**

We will recruit a cohort of 3,000 women newly diagnosed with breast cancer at hospitals in high (average, 20%) HIV prevalence areas, in Johannesburg, Durban, Pietermaritzburg, and Empangeni. At baseline, we will collect information on demographic, behavioral, clinical, and other factors related to access to health care. Every 3 months in year 1 and every 6 months thereafter, we will collect interview and chart data on treatment, symptoms, cancer progression, comorbidities, and other factors.

We will compare survival rates of HIV-infected and uninfected women with newly diagnosed breast cancer and their likelihood of receiving suboptimal anticancer therapy. We will identify determinants of suboptimal therapy and context-specific modifiable factors that future interventions can target to improve outcomes. We will explore molecular mechanisms underlying potentially aggressive breast cancer in both HIV-infected and uninfected patients, as well as the roles of pathogens, states of immune activation, and inflammation in disease progression.

**Conclusion:**

Our goals are to contribute to development of evidence-based guidelines for the management of breast cancer in HIV-positive women and to improve outcomes for all patients with breast cancer in resource-constrained settings.

## INTRODUCTION

Until recently, most malignancies in low- and middle-income, HIV-endemic regions of sub-Saharan Africa were related to infectious agents, such as Epstein-Barr virus and human papillomavirus. In the past decade, increases in longevity as a result of Westernization and the rollout of antiretroviral therapy (ART) have increased cancer incidence and shifted the spectrum of cancer diagnoses^1,2^ toward the epithelial malignancies that are typical of developed countries.^3^

Little is known about breast cancer (BC) outcomes in HIV-positive patients.^4,5^ In developed countries, BC may be slightly less common among HIV-infected than HIV-uninfected women or the general population^6-10^ because reproductive factors associated with HIV infection, such as young age at first pregnancy and multiple pregnancies, are associated with reduced risk of BC. In southern Africa, however, HIV may be associated with lower parity.^11^ If HIV-infected women have fewer children at an older age and breastfeed less than uninfected women, they may have higher risks of BC. HIV-related CD8+ T-cell dysfunction^12-17^ may affect cancer prognosis^18-24^ because CD8+ T cells can develop cytotoxic responses to tumor cell surface proteins. Although ART seems to mitigate the adverse effects of HIV on survival in certain cancers,^25,26^ its effects on breast cancer outcomes are unknown.

In a retrospective cohort of 220 BC cases in Uganda, where more than 80% had stage III or IV disease and 11% were HIV positive,^27^ HIV-infected patients had twice the 1-year mortality of uninfected patients. However, this study included only 24 HIV-positive patients with BC and lacked detailed information on their CD4 counts, viral loads, and ART use.

In 2010, South Africa had an age-standardized BC incidence rate of 25.86 per 100,000 women,^28^ but rates varied by ethnicity (Table 1). As of 2013, HIV prevalence among adults in South Africa was estimated to be 19.1% overall,^29^ but varied by age group, ethnicity, region, and sex; the highest HIV prevalence among sex and age groups in the general population was in women 30 to 34 years old (36%), but prevalence in female sex workers was nearly 60%.^30^

**Table 1:**
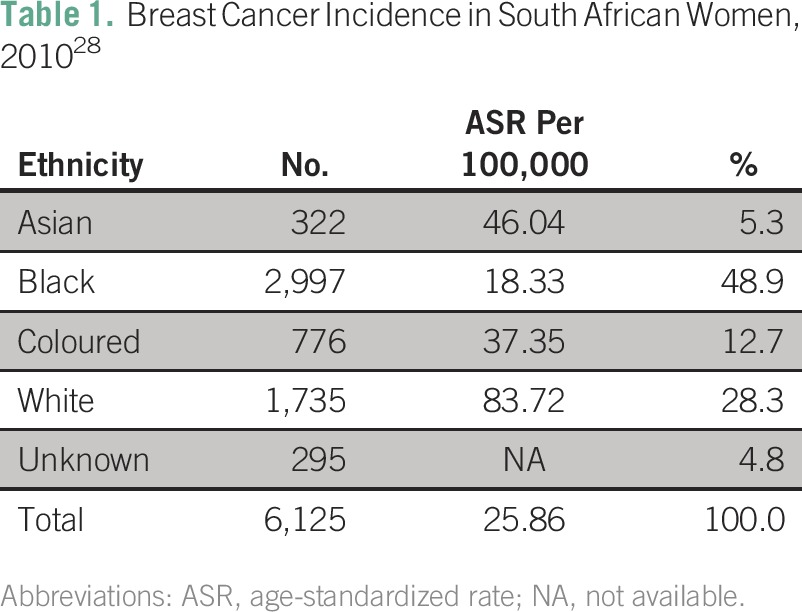
Breast Cancer Incidence in South African Women, 2010^28^

Although HIV prevalence and ART availability are similar in several sub-Saharan African countries, in most such countries, > 75% of BC is diagnosed at stage III or IV. In addition, receptor testing is not routinely available and treatment, where available, is often not affordable. Few countries have cancer registries or systems for reporting mortality data or detailed clinical and pathologic data of patients with cancer at diagnosis.^31^

South Africa has a pathology-based registry^32^ that reports incident cases and annual incidence rates overall and is stratified by population subgroup. The registry does not break down incident cases by clinical categories, such as stage or molecular subtype, nor does it report mortality data. In 2011, new legislation made cancer a notifiable condition, and the staffing of the registry was strengthened. Since then, the collection of public and private pathology data has been reliable and consistent. The registry is currently piloting a population-based cancer reporting system in a district east of Johannesburg, with a population of 3 million. However, rapid progression of disease, poor access to the health system, and rare diagnoses remain barriers to case finding.

In South Africa, cancer surgery, chemotherapy, radiotherapy, and hormonal therapies are available in tertiary referral hospitals; treatment costs to patients in the public sector are low or can be waived depending on patient resources; and social support mechanisms (eg, Cancer Association of South Africa care homes, hospital transport) may facilitate repeat hospital visits during treatment. Routine diagnostic work-ups include imaging; histologic confirmation conducted by the National Health Laboratory Service; immunohistochemistry (IHC) classification by estrogen receptor (ER), progesterone receptor (PR), human epidermal growth factor receptor 2 (HER2) status; and Ki-67 proliferation index staining on pretreatment biopsy or resection specimens.^33^

In a US cohort of patients with BC, triple-negative breast cancer (TNBC), characterized by a lack of ER, PR, and HER2 receptors,^34^ was found in 10% to 20% of newly diagnosed patients and was associated with poor outcomes.^35^ African patients with BC have been reported to be two to three times more likely to have TNBC than patients of European ancestry.^36,37^ However, in our recent Soweto (Johannesburg, South Africa) study, only 20.7% of black women had TNBC.^38,39^ Large-scale molecular subtyping studies of BC have been conducted, but mainly in US populations.

Increasing evidence indicates that patients with BC who receive the full course of prescribed chemotherapy or hormonal therapy fare better than those who receive incomplete therapy.^40-42^ HIV-infected patients may have fewer financial resources, less social support, and lower baseline WBC counts than uninfected patients.^43^ HIV-infected patients receiving chemotherapy may also have higher risk of myelosuppression as a result of poor bone marrow reserve, opportunistic infections, and interactions with ART.^44-46^ Such patients may receive insufficient anticancer treatment.

South Africa’s unique combination of high HIV prevalence, widely available ART, and advanced facilities for BC diagnosis and treatment in the public sector has motivated us to undertake the South African Breast Cancer and HIV Outcomes (SABCHO) study.

## PRELIMINARY DATA

In 2006, the Surgical Breast Unit at Chris Hani Baragwanath Academic Hospital in Soweto established a clinical database that now includes almost 2,000 patients. Baseline characteristics of the patients diagnosed before 2013 have been described.^39^ All patients are offered HIV testing during their diagnostic work-up, and women who test positive for HIV are referred to the specialist HIV unit so that they can begin ART before cancer treatment.

Of 1,092 consecutive patients with BC enrolled between 2006 and 2012, 765 (70%) were tested for HIV; 151 (19.7%) tested positive (including one-third of patients < 50 years old) and 37 (24.5%) had CD4 cell counts < 200 cells/L (median CD4 count, 316 cells/L).^38^ More than half of the patients were diagnosed with stage III or IV disease. Older age and residence farther from the hospital were associated with later-stage disease at diagnosis.^47^ HIV-positive patients with BC were younger than their HIV-negative counterparts, reflecting the age distribution of the HIV-positive population in the region, but did not differ in tumor characteristics.^38^

These findings raised questions about the pathways by which patients arrived at their BC diagnoses, the adjuvant treatment they received, and their ability, especially given HIV and other comorbidities, to tolerate chemotherapy and radiotherapy to the extent that they received it.

## METHODS

### Overall Study Design

**Fig 1:**
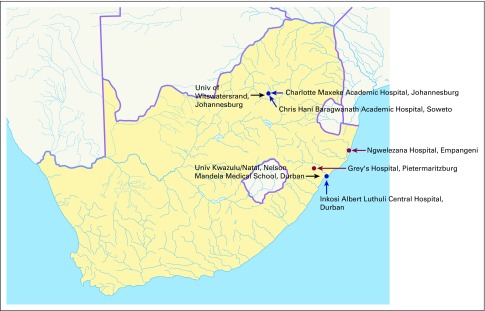
Locations of South African Breast Cancer and HIV Outcomes study sites.

For the SABCHO prospective cohort study, we will recruit 3,000 women with newly diagnosed BC at five hospital sites that serve populations with high HIV prevalence (Fig 1) over a 3-year period and follow them for at least 2 years. The study has been approved by the ethical review committees of the participating institutions.

### Study Sites

The key characteristics of each hospital are summarized in Table 2. Each site is the main public tertiary hospital and sees most women diagnosed with BC in its catchment area. Most sites primarily serve low- to middle-income black African populations with limited or no health insurance. The five sites diagnose up to 1,200 patients per year with BC; approximately 20% may be infected with HIV.^47a^

**Table 2:**
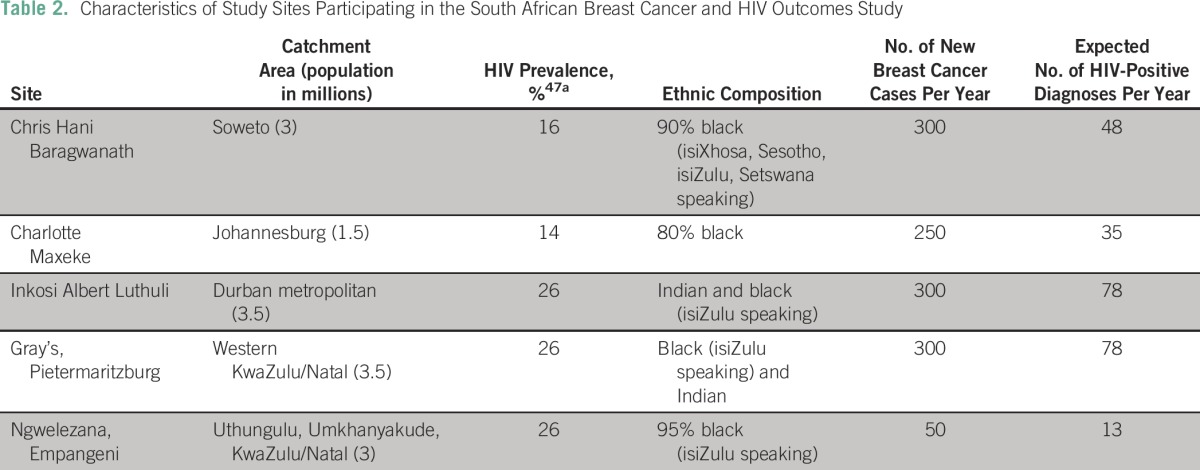
Characteristics of Study Sites Participating in the South African Breast Cancer and HIV Outcomes Study

### Study Population

#### Subject eligibility.

Female patients of any ethnicity and HIV status are eligible for study participation if they are ≥ 18 years of age, newly diagnosed with primary invasive BC at one of the participating hospitals, have no significant cognitive deficit, and have no previous cancer diagnosis other than nonmelanoma skin cancer or in situ cervical cancer.

As part of their routine diagnostic work-up, all patients are offered and almost all consent to HIV testing. Those who test positive are referred to the HIV unit so that they can initiate ART according to The South African Antiretroviral Treatment Guidelines 2013^48^ before cancer treatment. Patients may refuse HIV testing but consent to participate in our study; they will be categorized in analyses as HIV status unknown.

#### Recruitment.

At each participating hospital, a dedicated study nurse approaches every patient being seen for suspected BC, and obtains her demographic data and contact information. At the diagnostic confirmation visit, or by telephone if the patient does not return, the study nurse invites the patient to participate. If the patient agrees, a study nurse interviews her immediately or as soon as is feasible, before initiation of systemic or local therapy.

### Study Procedures

#### Baseline visit.

At the baseline interview (Table 3), the patient provides written informed consent for the study procedures, including administration of baseline and follow-up questionnaires, collection of a blood specimen, and use of the biopsy specimen and data from medical records. The patient may sign a separate consent for use of HIV-related data. Our validated Barriers to Care questionnaire (available on request) includes questions on BC knowledge and attitudes, social support, entry into the health care system, sources of delay in obtaining care, and facilitators of access to care. The study nurse obtains data on age, reproductive history, smoking, alcohol use, family history of breast and other cancers, and prior medical conditions from the baseline interview questionnaire and medical records. From the routine diagnostic work-up database, the nurse obtains the clinical stage; volume and grade of tumor; specimen margins; IHC data on ER, PR, and HER2 status (with fluorescence in situ hybridization reports for IHC 2+ results); and Ki-67 proliferation index, measured by the National Health Laboratory Service, Johannesburg, South Africa.

**Table 3:**
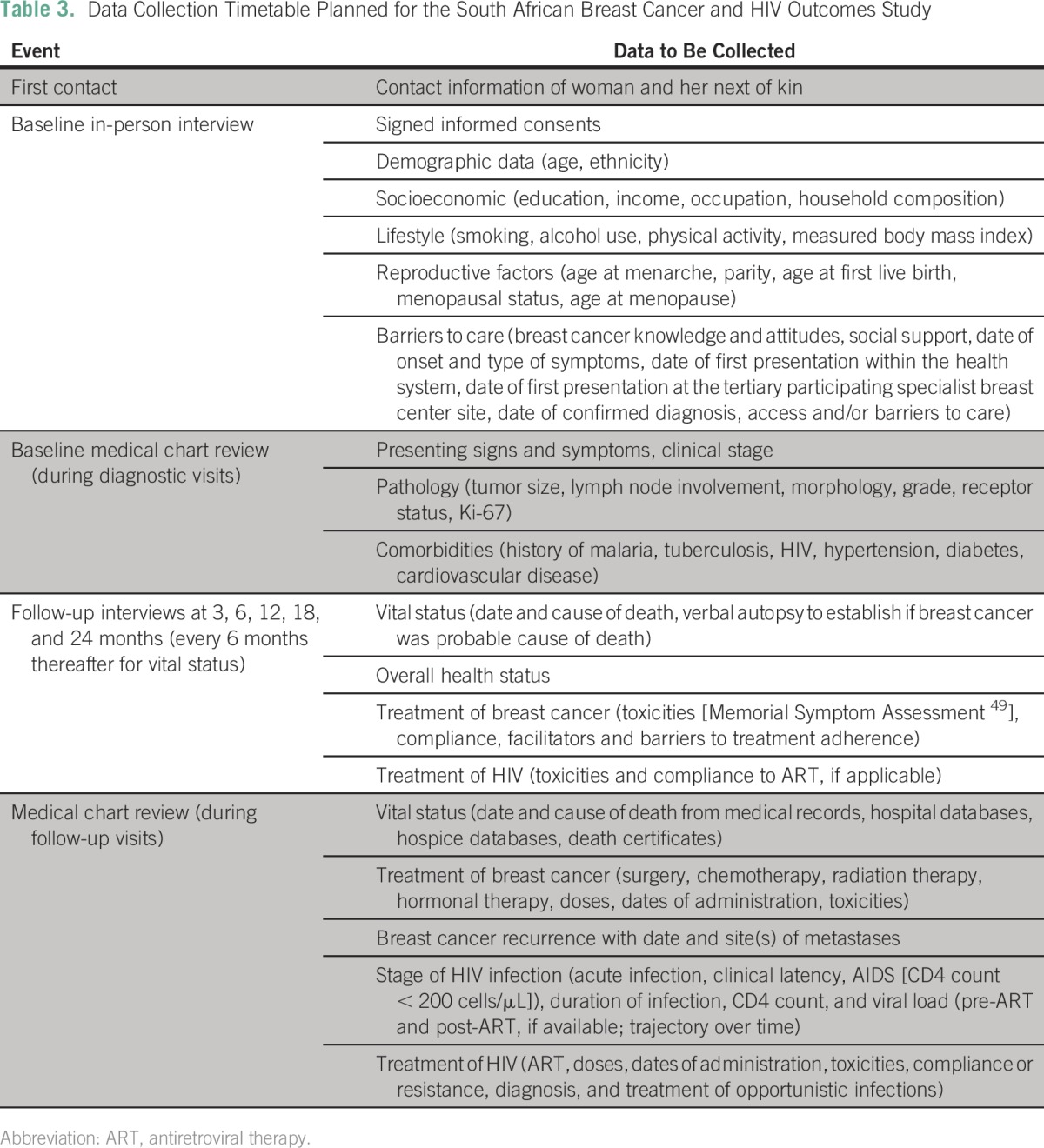
Data Collection Timetable Planned for the South African Breast Cancer and HIV Outcomes Study

For consenting patients who have HIV, the study nurse ascertains the stage of HIV infection (acute infection, clinical latency, or AIDS [CD4 count < 200 cells/μL]), as well as ART use, pre- and post-ART viral load (if applicable), and diagnoses and treatment of opportunistic infections.

#### Follow-up interviews.

Follow-up interviews will be conducted at 3, 6, 12, 18, and 24 months and every 6 months thereafter. The follow-up interview questionnaire (adapted from the Memorial Symptom Assessment)^49^ will cover self-assessed health status and treatment-related adverse effects. After 24 months, the follow-up interview will involve a clinical examination for signs of recurrence and will focus on performance status and vital status. If the patient has died, the next of kin will be asked for the date of death, the likely cause of death (on the basis of the 2012 WHO verbal autopsy instrument), and the impact of the patient’s death on the family.

Medical record reviews will be used to obtain data on the patient’s surgery type (breast conserving, mastectomy, axillary lymph node dissection) and timing (primary or adjuvant), as well as chemo-, hormonal, and radiotherapy dates, doses, toxicities, and compliance. Routine laboratory tests during chemotherapy (eg, CBCs, liver and renal function tests) will be reviewed to determine myelosuppression, renal and liver toxicity, and so on. Similar interview and HIV clinic record extraction methods will generate data on initiation, receipt, toxicity experiences, adherence to, and changes regarding ART regimens for HIV-positive patients. Typically, adherence/resistance is assessed by viral loads at 6 and 12 months after initiation of ART, then yearly; we will follow that protocol.

#### Biomarker assays.

We will obtain 10-μM tissue sections from 400 well-annotated BC cases (200 HIV positive and 200 HIV negative). From each tumor block, our pathologists will obtain a hematoxylin and eosin–stained slide to verify tissue morphology and tumor volume, and to guide macrodissection of the tumor to avoid normal tissue contamination, before mRNA extraction for the *PAM50* gene test using a NanoString assay.

The genomic NanoString assay has been found comparable to real-time reverse transcriptase polymerase chain reaction and DNA/RNA microarrays in its ability to profile hundreds of DNA and RNA samples with high sensitivity and precision. It requires fewer steps and is relatively easy to analyze and interpret. The protocol eliminates enzymatic reactions that introduce bias into the results. Therefore, it reduces human error and provides more accurate data on gene expression from mRNA extracted from formalin-fixed paraffin-embedded (FFPE) tumor samples. We will use the *PAM50* NanoString assay, as customized by Perou et al,^50^ for intrinsic subtyping and risk of recurrence analyses by *PAM50* and IHC4 methods.^50,51^ We have acquired the resources to conduct these assays at the University of the Witwatersrand.

Breast tumor development involves complex interactions among many cell types. Cytokines and chemokines secreted by tumor cells can promote or inhibit tumor progression. We will use blood samples collected from the patients to profile cytokine and chemokine expression using a multianalyte screening strategy by Luminex (Bio-Plex Pro cytokine assay, Bio-Rad, Hercules, CA).

### Analytic Approach

**Fig 2:**
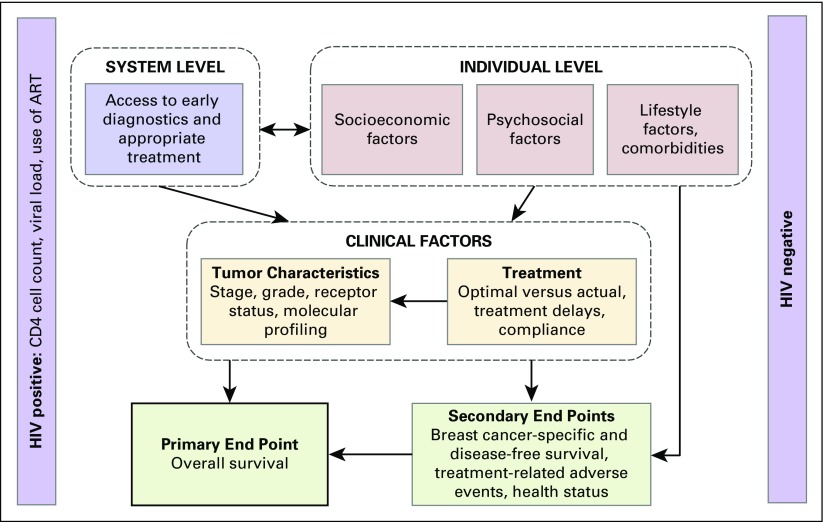
Causal pathways under investigation within the South African Breast Cancer and HIV Outcomes study for breast cancer survival among HIV-positive and HIV-negative women. ART, antiretroviral therapy.

We will use a classic bio-psychosocial theoretical model^52,53^ (Fig 2) to elucidate how system-level factors (access to early diagnostics and appropriate treatment), individual-level factors (socioeconomic status, psychosocial factors, lifestyle factors, comorbidities), and clinical factors (tumor characteristics, treatment, comorbidities) influence our primary and secondary end points among HIV-infected and uninfected patients with BC.

#### End points.

The primary end point is overall survival; secondary end points are BC-specific and disease-free survival, treatment-related adverse events, and health status (for the > 90% of women with stages I to III BC). Survival time will be calculated both from confirmed diagnosis and, as a sensitivity analysis, from the date of first presentation to a health care provider for a breast problem that is subsequently confirmed as BC to the date of death. Additional outcomes (Table 3) will be adherence to cancer (chemo-, radiation, and hormonal) and HIV therapy, as well as toxicities.

#### Statistical analyses.

The baseline data will be analyzed after recruitment is complete. We will compare HIV-infected and uninfected patients with respect to socioeconomic status, psychosocial factors, lifestyle factors, comorbidities, tumor characteristics, and surgical procedures, using *t* tests for continuous variables^54^ and χ^2^ tests for categorical variables to evaluate statistical significance.^55^

For survival analyses, we will use statistical methods described by Swaminathan and Brenner,^56^ including specific methods for estimating cancer survival in limited-resource settings where follow-up through electronic registries is often not possible. We will use similar approaches to analyze all time-to-event outcomes, including time between BC diagnosis and death; first clinical presentation and diagnosis; diagnosis and first treatment (any); surgery and systemic therapy; and diagnosis and BC recurrence. We will use medians and interquartile ranges to summarize the data. We will generate Kaplan-Meier estimates, with the log-rank test at the 5% significance level, to compare overall, BC-specific, and disease-free survival by HIV status.^57^

We will then develop Cox proportional hazards models to investigate factors associated with the above survival outcomes.^58,59^ These analyses will involve univariate assessment of each factor for association with survival outcome at a conservative 20% significance level and forward multiple regression modeling, in which all factors significant at the univariate level will be considered for significance at the 5% level. We will subdivide HIV-positive patients on the basis of CD4 count (≥ 500, 200 to 499, and < 200 cells/L) and prior HIV infection and receipt of ART at the time of cancer diagnosis.

We will also investigate determinants of optimal therapy for BC among HIV-positive and HIV-negative women, seeking modifiable reasons for disparities in cancer care and clinical outcomes.

**Fig 3:**
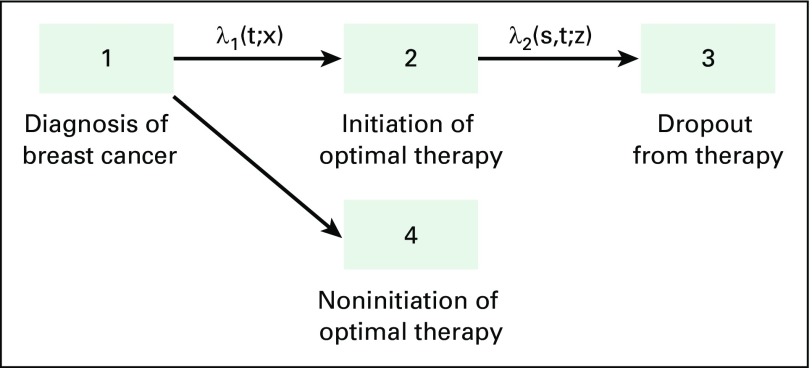
Scenarios for initiation of and adherence to breast cancer therapies. λ1(t;x) and λ2(s,t;z), hazard rate from state 1 to state 2 and from state 2 to state 3.

Figure 3 depicts our four-state stochastic process, where λ1(t;x) and λ2(s,t;z) are, respectively, the hazard rate from state 1 to state 2 and from state 2 to state 3; x and z are covariates that may be time dependent. State 1 is diagnosis. The time origin is the date of BC diagnosis. State 2 is cancer therapy (chemotherapy, radiotherapy, or hormonal therapy), and t is the date of the patient’s first cancer therapy. State 3 is the dropout state, and s is the dropout date. Patients who complete optimal therapy will be censored at the completion date. Patients who do not initiate optimal therapy within 6 months after diagnosis will be in state 4, which is noninitiation of optimal therapy. We will use logistic regression to analyze the association of HIV and covariates with noninitiation of therapy. We will also relate HIV and covariates to initiation time and dropout time using Cox proportional hazards models.

Using our NanoString data, we will classify each patient as luminal A, luminal B, HER2-enriched, basal-like, or normal-like, and as low, intermediate, or high risk.^60^ We will then compare the proportions of cases in each category in the HIV-positive and HIV-negative groups using χ^2^ tests. We will also generate a risk of recurrence (ROR) score from the *PAM50* test, which is derived from the gene expression profile (with special weighting of proliferation-associated genes) and accounts for tumor size.^61,62^ Two-sample *t* tests (or nonparametric analogs) will be used to compare mean ROR scores by HIV status. Results will be compared with those obtained from the cost-effective IHC4 ROR algorithm.

We will use the Kruskal-Wallis analysis of variance by ranks method to compare cytokine expression levels of the intrinsic subtypes in the context of HIV infection, and we will conduct multiple-comparison two-tailed post hoc tests to home in on differences between groups by the Kruskal-Wallis test. We will use hierarchical clustering methods to show the cytokine associations within each subtype and to generate dendograms, using the unweighted pair-group average and evaluating Euclidean distance to indicate similarities.

## RESULTS

Recruitment for the SABCHO study began in October 2015 and is in progress.

## DISCUSSION

This study expands on our previous data collection efforts by including long-term follow-up for survival, assessing detailed treatment-specific adherence and toxicities, and collecting similar data at five institutions in South Africa. In addition, it includes more rigorous phenotyping with the *PAM50* genomic assay.

The few genomic studies of breast tumors in African women have mainly used FFPE tissues and existing multigene tests to identify gene expression signatures on the basis of microarrays and real-time reverse transcriptase polymerase chain reaction applied to frozen tissue. These assays are technically complex, and their results may not be reproducible. Instead, we are using the nanotechnology-based nCounter digital gene expression platform, which has highly reproducible results with FFPE RNA samples. The system automatically applies a series of quality control metrics to each sample during analysis to determine whether results fall within expected values. We expect to measure gene expression in FFPE tumors accurately and to identify different spectra and frequencies of RNA transcripts.

In conclusion, the complex problem of HIV and cancer in resource-constrained settings calls for the prospective, integrated, multidisciplinary approach we will use in the SABCHO study. Our data will provide a basis for assessing health care system responses to patient needs and for the development of interventions to improve survival rates in South Africa and potentially other sub-Saharan African countries.

The strengths of our study include its focus on a region of sub-Saharan Africa with a high prevalence of HIV, a multiethnic population with poor BC outcomes, and facilities for efficacious diagnosis and treatment. To our knowledge, this is also one of the first large-scale studies of tumor molecular profiling among African patients with BC, using gene expression signatures with known prognostic significance and correlating them with HIV status. Our intention is that this multicenter study will help identify context-specific, modifiable factors that can be targeted in future interventions to improve BC outcomes in South and sub-Saharan Africa.
